# Management of Co-existing Dementia and Hearing Loss in Social Care Settings: A Focus Group Study

**DOI:** 10.1177/14713012251356010

**Published:** 2025-06-28

**Authors:** Emma E Broome, Alice Green

**Affiliations:** 1 Hearing Sciences, Mental Health and Clinical Neurosciences, School of Medicine, 6123 University of Nottingham, Nottingham, UK; 2 574111 National Institute for Health and Care Research (NIHR) Nottingham Biomedical Research Centre, Nottingham, UK; 3 School of Medicine, 6123 University of Nottingham, Nottingham, UK

**Keywords:** dementia, hearing loss, care homes, social care, qualitative research

## Abstract

**Background and Objectives:** Dementia and hearing loss are highly prevalent and increase in prevalence and severity with age. Hearing loss is often overlooked in people living with dementia, resulting in under-diagnosis and lack of appropriate management. Both conditions present substantial challenges for individuals and healthcare systems more broadly. The presence of both conditions can mask each other, presenting challenges for both diagnosis, treatment and support. The aim of this study was to qualitatively explore the experience, needs and opinions of how to manage hearing loss in people living with dementia in social care settings from multiple perspectives. **Research Design and Methods:** A qualitative study using focus groups with key stakeholder groups was conducted. Data were analysed using thematic analysis. Participants included seven social care professionals (aged 25-68), six informal carers (aged 56-92) and one person living with dementia and hearing loss (aged 69) (m = 21%, f = 79%). **Results:** Five themes were identified: (i) inclusion; (ii) communication, (iii) hearing aids, (iv) health services and (v) training of care staff. **Discussion and Implications:** Findings highlight the need for comprehensive training to help support the management of hearing loss in people living with dementia. Training on the use and maintenance of hearing aids would be particularly valuable for staff. Due to the progressive nature of both conditions, individuals in receipt of social care should be reviewed regularly to ensure that care needs are adapted to suit the progressive nature of the conditions.

## Introduction

The coexistence of dementia and hearing loss is common, both of which increase in prevalence with age. These conditions are associated with risks such as depression (Enache et al., 2011; Lawrence et al., 2020), social withdrawal (Kuiper et al., 2015; Weinstein & Ventry, 1982), increased frailty (Petermann-Rocha et al., 2020; Tian et al., 2021) and reduced quality of life (Dalton et al., 2003; Keating & Gaudet, 2012). There are similarities in the behaviours and symptoms of both conditions, for example understanding speech (Hardy et al., 2016), difficulties in social interactions, such as repeating questions or inappropriate word use (Burgon et al., 2023; Heffernan et al., 2022; Jorgensen et al., 2016; Ray et al., 2019) and communication difficulties (Crosbie et al., 2019). Given that both conditions may mask each other, distinguishing the cause of impairments in communication, function, and social participation is challenging (Ray et al., 2019). The prevalence of cognitive impairment is greater in people with hearing loss compared to those without (Huang et al., 2023; Lau et al., 2022). However, hearing loss is often overlooked in people with dementia, resulting in under-diagnosis and a high rate of untreated hearing loss and appropriate management (Jorgensen et al., 2016). Combined, these factors make it challenging for health and care services to provide appropriate identification, support and management of hearing loss in individuals living with dementia.

In the United Kingdom (UK), approximately 75% of care home residents have a degree of hearing loss, due to rise to 80% in the next 10 years (Royal National Institute for Deaf People, 2018), and an estimated 80% of care home residents have dementia (Prince et al., 2014). These figures indicate that a large proportion of care home residents will live with both conditions, making care and management more complex. A systematic review reported that hearing aids, provided for people with dementia living in the community, were effective in reducing hearing disability and the behavioural and psychological symptoms of dementia (Dawes et al., 2019). However, care homes present a unique challenge to the management of hearing loss in this population. Hearing aid use in care settings is typically poor (Cohen-Mansfield & Taylor, 2004a); reasons for non-use range from poorly fitting, devices becoming broken or lost, lack of access to replacement batteries, and residents relying on support from care staff to use and handle them (Cohen-Mansfield & Taylor, 2004b; White et al., 2021). Lack of hearing aid use may heighten communication difficulties which intensifies particular dementia behaviours (Crosbie et al., 2019). In addition, environmental and social factors within the care setting can hinder the application of appropriate hearing healthcare. As both conditions affect communication, interactions between staff and residents may be disrupted, potentially reducing the quality of care delivered (Ray et al., 2019).

A systematic review reporting on the effectiveness of hearing rehabilitation for care home residents with dementia, demonstrated that although residents benefited, this form of hearing healthcare was not always prioritised (Cross et al., 2022). Similar findings were highlighted by Crosbie et al. (2019) in a realist review exploring factors which contribute to the management of hearing-related communication in care homes. Their analysis suggested that leadership and quality training in dementia and hearing loss were important to prompt effective hearing-related communication.

The aim of this study was to qualitatively explore the experience, needs and opinions of how to manage hearing loss in people living with dementia in social care settings from multiple perspectives (people with lived experience of dementia and/or hearing loss, informal carers and social care professionals).

For this study, social care settings were defined as settings which provide care for people who require it provided in the community (e.g. community nursing), residential (e.g. care homes or nursing homes), or domiciliary settings (e.g. home care) (The King’s Fund, 2017; Thorlby et al., 2018). This approach enhances understanding of the challenges faced supporting hearing care needs in people living with dementia by providing in-depth examination into key stakeholder perspectives, experiences and practices in real world settings for example care homes.

## Research Design and Methods

### Design

This study used a qualitative approach to examine the management of hearing loss in people living with dementia in social care settings in depth and detail. It is reported using the Standards for Reporting Qualitative Research (SRQR) (O’Brien et al., 2014).

### Participants and Recruitment

People with lived experience were eligible if they were aged >18 years and had a diagnosis of or were receiving care as someone living dementia and/or a hearing condition (e.g. hearing loss). This approach accounted for the gap between estimated and recorded dementia diagnoses (Prince et al., 2011), with over one third of individuals potentially living with dementia remaining undiagnosed but receiving formal care (Alzheimer’s Research UK, 2025). Informal carers (e.g. family members) were eligible to take part if they provided support for someone living with dementia/mild cognitive impairment and/or hearing condition. Social care professionals (SCPs) were included if they were aged >18 years and were employed within a health or social care setting in the UK as per the definition of social care setting above. This included a broad range of roles such as care home staff, domiciliary care workers, social workers, and others involved in the delivery of social care services to individuals living with dementia and hearing loss.

Participants were recruited purposefully ensuring a representative sample with a wide range of experiences (Creswell & Poth, 2018) to allow for divergent perspectives. This sampling method also enabled the triangulation of qualitative data from multiple perspectives about the management of hearing loss in people with dementia (Lincoln & Guba, 1985). Focus groups allow for interactions among participants, enabling the exploration and elaboration of opinions that may not have arisen outside of the group (Bender & Ewbank, 1994; Kitzinger, 1995).

People with dementia and hearing loss, and their informal carers were recruited through the NIHR Nottingham Biomedical Research Centre’s research participant database and social media posts. SCPs were recruited through professional contacts of the research team and social media posts.

Ethical approval was issued by the University of Nottingham, Faculty of Medicine and Health Sciences Research Ethics Committee (REF: FMHS 438-0122) on September 16^th^ 2022. All participants provided electronic informed consent.

### Procedure

A participant information sheet was emailed to individuals who expressed an interest in the study, explaining the background and aims of the study. Demographic information was collected via a brief online survey. Focus groups were held online using Microsoft Teams, enabling the inclusion of participants from around the UK and accommodating the schedules of SCPs from different care settings. Focus groups with SCPs took place separately to other contributors.

All focus groups were conducted by the lead researcher [EB], and a medical student conducting research for an honour’s thesis project at the time of data collection. The lead researcher had experience running focus groups and interviews with older adults. Several adaptions were made to facilitate the participation of people with dementia and hearing loss during the online focus groups. For example, the focus group sizes were kept small and we encouraged the use of communication aids such as live captioning and the raise-hand function to minimise overlapping speech. Both researchers were female and had no prior relationship with any of the participants. The focus groups were video-recorded, transcribed verbatim, and anonymised during transcription; transcripts were not reviewed by participants. Each participant was given an identification code for illustrative quotes (e.g., SCP 1).

The researcher explained the aim of the study and the procedure and answered any questions from participants. The topic guide (see supplemental material) included broad questions to create open conversation and generate comprehensive data; it was used flexibly to facilitate discussion, prompts were used to explore participants perspectives, experiences and practices. Open-ended questions allowed participants to describe their experience, obtain diverse perspectives and thus reduce bias (Creswell & Poth, 2018). The topic guide was adapted throughout data collection to gain deeper insights into subjects introduced by participants. Both researchers kept a reflexive diary throughout the data collection process to enhance credibility (Fade & Swift, 2011) and held debriefing meetings about the content of each focus group on completion.

### Data Analysis

An inductive thematic analysis was conducted on the transcripts using NVivo (version 12) software. The six phases of thematic analysis were followed: (i) data familiarisation included re-reading transcripts, (ii) generation of initial codes by examining the data as a whole with salient features coded in a systematic fashion; (iii) searching and grouping codes into themes including all relevant data, (iv) reviewing themes based on their conciseness and relevance, (v) defining and naming themes clearly and (vi) writing the narrative with illustrative, relevant examples relating to the research aim (Braun & Clarke, 2006).

No theoretical framework was used for this analysis; themes were derived from the data using open coding. The data were coded completely by the second author [AG]. Regular peer-review meetings between the researchers were held to review the codes, the discussions resulting in merging and dividing some groups which were then grouped into categories and themes. This form of peer assessment enhances the rigour of the qualitative analysis (Anney, 2014; Krefting, 1991).

## Results

Six focus groups were conducted between October–November 2022, comprised of one person with dementia and hearing loss, six informal carers and seven social care professionals (SCPs). Each participant attended one focus group only, with group sizes ranging from one to four participants. The average length of a focus group was 48 minutes (range: 35-60 minutes).

The demographics of recruited participants are reported in Table 1. Across all focus groups, the majority of participants were female (n = 11) and were White British (n = 12). The mean age of person with dementia and hearing loss /informal carers was 65.7 years (SD 11.4). Of the SCPs recruited, the mean age was 37.5 years (SD 13.2). The length of time in their role ranged from <2 -10+ years. The type of social care setting SCPs worked in included specialist dementia care homes (n = 5), nursing home (n = 1) and one participant who had worked in both types of care homes.

**Table 1.** Participant Demographics

	Informal carers (*n* = 6)	PWDHL (*n* = 1)	Social care professional (*n* = 7)
Age			
Mean, (SD), [range]	65.2 (±13.4) [56–92]	69	37.7 (±14.3) [25–68]
Gender			
Female	4 (66.7%)	0	7 (100%)
Male	2 (33.3%)	1 (100%)	0
Ethnicity			
Asian or Asian British	0	0	1 (14.3%)
Black or Black British	0	0	1 (14.3%)
White British	6	1	5 (71.4%)
Employment status			
Full time carer	2 (33.3%)	0	0
Full-time employed	1 (16.7%)	0	4 (57.1%)
Part-time employed	2 (33.3%)	0	2 (28.6%)
Retired	1 (16.7%)	1	1 (14.3%)
Job role			
Activity coordinator			2 (28.6%)
Care assistant			2 (28.6%)
House leader			1 (14.3%)
Senior care assistant			1 (14.3%)
Sensory social worker and care manager			1 (14.3%)
Years working in care home (years)			
<2			1 (14.3%)
2–5			2 (28.6%)
6–10			2 (28.6%)
10+			2 (28.6%)

### Qualitative findings

Five themes were identified from the thematic analysis: (i) inclusion; (ii) communication; (iii) hearing aids; (iv) health services; and (v) training of care staff.

The last two focus groups did not result in any new themes or sub-themes, indicating that data saturation was reached as the data collected repeated what was expressed in previous data (Saunders et al., 2018).

### THEME 1: Inclusion

#### Stigma

The perceived stigma of both dementia and hearing loss was reported to affect how people with dementia and hearing loss are included in everyday life. Some participants felt that people with dementia and hearing loss are treated with *“complete disregard”* (Informal carer 2). It was reported that healthcare professionals often deferred questions to carers rather than the people with dementia and hearing loss, contributing to feelings of exclusion. Generally, it was felt that there is a lack of understanding about both conditions, this influenced the extent to which people felt stigmatised. Some informal carers described how healthcare professionals compensate for the communication difficulties encountered by “*just shouting”* (Informal carer 5). Others described that one way to overcome the stigma and negative preconceptions encountered is by becoming their own advocate:

[If] I don’t understand anything, I say “Excuse me doctor, I do not understand I need you to explain it”. Person with dementia and hearing loss 1

However, the impact of consistently having to advocate for their relative contributed to the burden felt by informal carers:

I’m here but the person inside is exhausted and worn out and running out of steam so there is only so many drums I can bang. Informal carer 2

#### Isolation

The experience of isolation and social withdrawal was explored in all focus groups and related to the inclusion of people with dementia and hearing loss in activities within residential care setting:

It is a very isolating experience to have people around you but not be able to engage with them or communicate with them properly. SCP 6

Informal carers described how they felt that relatives were excluded from organised activities within the care home due to their hearing loss. It was felt that social activities not adapted for hearing loss would provide little benefit, thus contributing to social isolation. Reference was made to the use of music as a therapy tool used within the care settings; however, there were mixed responses regarding its benefit for people with dementia and hearing loss. For example, participants described how some residents could participate despite their hearing loss, whereas others reported that residents did not engage.

They can still sometimes follow it even though they can’t hear it if they are all clapping. SCP 7

### THEME 2: Communication

#### Impact on communication

The impact of the symptoms associated with the two conditions on an individuals’ ability to communicate, and consequently their quality of life was recognised by all participants:

It’s like communication problem on top of communication problem. SCP 6

The identification of symptoms, formal diagnoses, and management of either condition was made more challenging, due to symptoms masking each other. It was noted by SCPs that people with dementia don’t “*necessarily have the capacity to tell us all of these things*” (SCP 5) in relation to impairments which progress over time. Both SCPs and informal carers described the difficulty of recognising which behaviours were caused by which condition:

I really don’t know if that’s to do with a behavioural issue with the dementia, or if it's to do with the hearing, or if it’s a mixture of both, but it's difficult. Informal carer 2

Sometimes he shuts down because he doesn’t want to communicate with you, and sometimes he doesn’t hear what you’re saying, and the difference between the two isn’t something that's always discernible. Informal carer 2

SCPs disclosed that they felt people with dementia and hearing loss often took longer to process information, making conversations disjointed and slow. The communication challenges experienced negatively impacted SCPs relationships with their residents, resulting in residents becoming withdrawn and unhappy due to a lack of autonomy:

If we are forcing the residents to do this, eat this, drink this, with the passage of time they keep their mind, their mouth, their psychological level, and physical activities down, because the workers only force them, instead of having good communication with them. SCP 2

#### Barriers to communication

While all participants noted the impact of dementia and hearing loss on communication, only SCPs identified specific barriers relating to communication. They discussed the communication challenges associated with mask wearing, noting how the masks created a visual barrier to social cues:

Because the mask is on, they can’t see you smiling then they can’t hear you. It just puts that barrier in place. SCP 3

SCPs reported that mask wearing affected individuals who rely on lip-reading as a supplemental communication strategy:

When we’re still wearing the masks, it makes such a huge impact on the communication side of things, people rely on those lip movements. SCP 5

The physical barrier created by masks was stated to cause some resident’s distress. Talking about this issue one participant said: *we have one lady who is so distressed she even pulls your mask off* (SCP 5).

Background noise present in residential care homes was another barrier, which at times caused distress in people with dementia and hearing loss:

When you’re trying to talk to them and they’ve got the potato peeler going in the background, it causes behaviours because they can’t hear you. SCP 3

Issues relating to the auditory landscape of the care home were prominent. The care home setting was described as “*overpowering*” (SCP 3) which disrupted residents’ communication with staff and family members. SCPs felt that the impact of the noisy environment exacerbated some of the behavioural symptoms associated with dementia. SCPs highlighted the challenge of adapting communal areas and activities to support individuals with different sensory needs. A small number of SCPs reported how their care setting had different communal areas for people at various stages of their dementia journey. Quieter areas were typically available and utilised by individuals who had more severe dementia; however, there was no indication from participants data that these spaces were specifically used in response to sensory needs related to hearing loss.

#### Communication techniques

SCPs identified several communication techniques they implemented to combat the barriers faced when interacting with people with dementia and hearing loss. Simple ideas included getting closer to the person and being “*very patient and visual*” (SCP 7), along with “*making sure they can see your face*” (SCP 7). SCPs also suggested more structured solutions such as using a whiteboard to communicate, which they felt was effective in those with severe hearing loss:

We used to have one gentleman who was very deaf…he could understand writing so you could…write very simple things like, do you want tea or coffee, something like that and he’d be able to read it and then reply back to you. SCP 5

Allowing individuals to make choices by using visual cues was described by staff as a way to support autonomy, whilst also facilitating better communication between staff and people with dementia and hearing loss. However, these techniques were described as less effective as the dementia progresses over time, meaning that strategies needed to be adapted:

We used a whiteboard and we used to communicate via writing things down, he would read that. But then as his dementia journey progressed, it became really, really difficult because the confusion came in, he wasn’t really able to read it. SCP 5

Suggestions of other techniques such as a “*set of visual aids or something like that posted up in the deaf person with dementia’s room which says: remember just stand in front of me before you talk remember not to have noisy sounds going on and remember that I tend to withdraw*” (Informal carer 4), were proposed as a reminder for staff members to be aware of how everyone has different communication needs.

### THEME 3: Hearing Aids

#### Importance and value of hearing aids

Informal carers highlighted the importance of hearing aids for people with dementia and hearing loss, describing them as an “*essential tool*” (Informal carer 2) and *“life changing”* (Informal carer 6). SCPs also highlighted the positive impact that hearing aids had on the ability of people with dementia and hearing loss to communicate. When hearing aids were used, SCPs felt they didn’t need to raise their voice, and improved communication was noticed:

[When] they’ve got the hearing aid in…then that person’s going to be able to communicate with us better. SCP 7

Most participants felt that, in those who would tolerate them, hearing aids were associated with a significant positive effect on quality of life and communication.

#### Tolerance of hearing aids

Tolerance of hearing aids in PWD was highlighted as a particular challenge. Most SCPs reported that people living with dementia and hearing loss would not accept wearing hearing aids. Both SCPs and informal carers recounted occasions where individuals refused to wear their hearing aids, forgot to put them in, or would take them out. Some SCPs described instances where people with dementia and hearing loss would become aggressive when they attempted to promote hearing aid use. Despite this, it was considered important to all participants, that a person-centred approach was implemented when providing hearing aid management for people with dementia and hearing loss. Factors such as, knowing the person, and their individual tolerance to hearing aids was deemed valuable knowledge:

You get to know each person individually, so you know who has hearing aids, who will wear them. SCP 3

However, there was a tension between the views of SCPs and informal carers on how hearing aid use should be supported. SCPs felt that family carers were advocates wearing hearing aids continuously, whereas they themselves acknowledged that that may not always be what the person wants:

My experience has been that it’s family that want the people to keep their hearing aids in and the actual clients themselves rarely wear them. SCP 4

Informal carers described how their relatives had difficulty in both maintaining and using their hearing aids. Common complaints included hearing squeaking noises from their hearing aids, a sign they are not working correctly, or that they were difficult to fit. In addition, some discussed the challenge of wearing both hearing aids and glasses, with the result often being refusal to wear one of them:

So wearing glasses with over the ear, [with] behind ear hearing aids is really, really uncomfortable, really tricky... My dad just won’t wear his glasses, refuses to wear his glasses and I think that’s because he’s got as I said, one over ear [hearing aid] now. Informal carer 6

#### Management and maintenance of hearing aids in care settings

The discussion surrounding management of hearing devices was varied, with SCPs suggesting challenges such as lost hearing aids, and some even ending up “*in the bin”* (SCP 7). One SCP, working in a care home, described how they had implemented an organisational system where each resident had a named container for their hearing aids, which was proving to be effective. The responsibility of hearing aid maintenance fell on SCPs, as many residents were not able to manage this aspect themselves due to limited dexterity:

They struggle just because they don’t know how to change the batteries and how the tubing needs to be. SCP 3

Despite this, SCPs reported that they had little knowledge of hearing aids and their maintenance. Indeed, one participant reported how hearing aid support in her workplace was reliant on others’ personal experiences:

One of our members of staff is registered deaf so that helps us because she supports us quite a lot and we have one lady who’s got a child who wears hearing aids, so we suck on those skills and say come and show me and we try and utilise the skills we have already. SCP 5

Factors which affected the ability of SCPs to implement hearing aid use included high workload and lack of staff; the management and maintenance of hearing aids were not always a priority.

### THEME 3: Health Services

#### Access to audiology services

SCPs reported that compared to other health services, such as GPs, opticians and dentists, there was a lack of presence of audiology services within residential care home:

I think it’s the hearing side of it that isn’t there really... we have regular GP’s, we have regular opticians, we even have the dentist quite a lot. SCP 5

Indeed, SCPs described the support their residents received from audiology services as minimal and impersonal. Their perception was that care home residents do not receive the same level of support from audiology services as people living in the community. For example, it was reported that residents were unable to get their hearing aids moulded to their ears:

Even with broken hearing aids themselves, they just get sent off. They don’t even think about like the moulds of the ear or anything anymore. SCP 5

Many participants complained of the long wait times to access National Health Service (NHS) hearing services, in one instance an informal carer reported having to wait four months to receive their relative’s hearing aids. Some informal carers described how they felt they had to be pro-active in order to access hearing services, for example by taking their relatives hearing aid tubes to the hospital themselves and waiting for replacement parts.

#### Dementia pathways

Most informal carers were not aware of any dementia specific pathways within audiology services. Generally, it was felt that there was a lack of support for people with dementia and hearing loss within the healthcare system. SCPs highlighted the need for greater support for older people living with comorbid conditions. Services which did have dementia awareness appeared to provide better support. An informal carer who obtained private hearing aids for their relative living with dementia reported a positive experience interacting with a healthcare professional who had dementia training. Similarly, the participant with lived experience of dementia and hearing loss stated that they had been well supported and felt comfortable returning to the audiology service when necessary, however they acknowledged that this was in part due to their own advocacy:

I pointed everything out to my audiologist when I went and I said, look, I’ve got dementia you will need to explain things to me in a slow if I don’t understand I’m going to ask. Person with dementia and hearing loss 1

Informal carers described the struggle of obtaining a formal diagnosis from a healthcare physician as “*dementia and hearing loss are both hidden”* (Informal carer 4). This topic was reflected by SCPs, they described how many of their residents’ displayed symptoms of dementia and hearing loss, but the perceived severity of dementia prevented a formal diagnosis of hearing loss being made:

We’re at a later stage of their dementia journey, but like I said before, actually they just can’t see, and they just can’t hear very well and that is where the misdiagnoses are coming from. SCP 5

### THEME 4: Training of Care Staff

This theme relates to the lack of formal training that SCP receive to support people living with dementia to manage their hearing loss. The amount and quality of training reported by SCP was varied, however there was consensus in that there was lack of training on how to support hearing loss in general, particularly in individuals living with dementia. Most SCPs had not received any formal hearing aid maintenance training, despite caring for many individuals living with hearing loss:

If we talk about dementia and hearing loss, two particular things, they have the lack of training in the proper system. SCP 2

The one thing we didn’t look at was the maintenance of the hearing aid. SCP 4

Two participants described how they relied on their own personal experience of hearing loss to support residents with hearing aid maintenance:

I know how to put the hearing aids in but that’s not because someone trained me, that’s because I’ve learned how to do it myself. SCP 7

Other participants, who had no personal experience or formal training in hearing loss, struggled with this aspect of hearing care for their residents. This was identified as a gap in staff training:

I can identify this as a hearing aid, but I wouldn’t be able to put one in, I wouldn’t be able to clean one. I wouldn’t know how to change the battery or turn it on and off I have no idea because I’ve never experienced it and I don’t know anyone that has them, like in my family. SCP 6

Both SCPs and informal carers highlighted the value of additional training to support hearing health in people living with dementia. One aspect which SCPs thought would be helpful is how to support and communicate with people with dementia and hearing loss at different stages in their dementia journey. They identified that communication and hearing needs varied depending on the individual and where they were in their dementia, and hearing loss journey. There was a shared desire for this gap in training to be filled, and the importance of connecting it to dementia training:

The hearing side of things is something that we are trying to push, we don’t have much on the hearing side at all apart from the basic how to put a hearing aid in and things like that. There’s no element of actual how to communicate correctly with these people at the different stages of dementia. SCP 5

## Discussion

This study explored the experiences of manging hearing loss in people living with dementia in social care settings from multiple perspectives. Overall, there appeared to be a lack of evidence-based strategies to support people with dementia to manage their hearing loss. Residents are reliant on support from staff to support hearing aid use (Cohen-Mansfield & Taylor, 2004b). In the present study, the absence of formal training on hearing loss and hearing aid maintenance, was identified as a significant barrier to providing effective hearing care. While the participants did not directly link this to low hearing aid use, the reported knowledge gaps suggest a potential impact on the quality and consistency of hearing care and support people with dementia and hearing loss receive.

Poor hearing is suggested to contribute to the social isolation of residents living with dementia and hearing loss (Pryce & Gooberman-Hill, 2012); this was identified by participants in the present study. Challenges associated with hearing aid use reported in this study, such as poor hearing aid fit, tolerance and hearing aids being lost or broken are consistent with the literature (Dawes et al., 2021). Participants universally agreed that hearing aids can be beneficial to people with dementia and hearing loss, improving communication and thus social inclusion. Hearing aid use has been associated with improved quality of life and reduced social isolation and depression in older adults (Chisolm et al., 2007). Despite this, participants described that tolerance to wearing hearing aids in those living with dementia was low; some SCPs reported increased agitation in people with dementia and hearing loss when encouraging them to use their hearing aids.

One issue that emerged from these findings was the tension between the perspectives on hearing aid use; informal carers advocated for prolonged use whereas SCPs felt hearing aid use depended on the resident. Previous research has indicated the benefits of shared decision making on residents, families and staff (Foundation, 2021) however, this appears to be reliant on the engagement and collaborative relationship between each group (Lynch et al., 2022). In this study, informal carers felt that open communication was key in order to avoid conflict between residents, informal carers and SCPs; this finding supports other qualitative studies where communication with mutual cooperation and shared expectations helped facilitate positive staff-family relationships (Bauer et al., 2014).

Our findings highlight the need for comprehensive training for SCPs to help support the management of hearing loss in people living with dementia. These results corroborate findings of other research in this area (Crosbie et al., 2019; Cross et al., 2024). Training on the use and maintenance of hearing aids would be particularly valuable for staff and would support the use of hearing aids in social care settings. SCPs in this study reported non-evidence-based methods for providing hearing aid support to residents such as learning from others personal experience. To remedy the lack of formal training in this area Cross et al. (2024) have developed a multi-component behaviour change intervention, using intervention functions such as education and training to help care staff provide hearing care to residents with dementia. The importance of training on hearing loss and dementia, managing noise in care homes, and monitoring hearing-related communication needs was also highlighted by Crosbie et al. (2019). It is worth noting that there was an appetite from all participants for this training gap to be filled, citing benefits for residents and staff alike, primarily by improving communication.

Another important finding was how participants emphasised the importance of recognising the heterogeneity of people living with dementia and hearing loss. The positive effects of providing person-centred care and treating people with dementia and hearing loss with empathy and positive regard have been highlighted in this area (Crosbie et al., 2019). Similarly, the findings in this study emphasised the benefits of knowing the people with dementia and hearing loss individually in order to provide appropriate and tailored care and support. To ensure care keeps up with the progressive nature of dementia and hearing loss, it is important for SCPs to be aware of the presence, and severity, of the conditions in each individual. The strategies SCPs rely upon to communicate with people with dementia and hearing loss did not appear to always be effective, particularly as residents’ dementia and/or hearing loss deteriorated. This further highlights the complications of managing coexisting dementia and hearing loss, as care must evolve alongside the progressing symptoms in an individual. Therefore, formal training for both conditions should be provided, focusing on monitoring and adapting the care needs of residents over time. Additionally, emphasis should be placed on care protocols regularly as this would facilitate the evolution of communication and management techniques appropriate to people at different stages of their condition. This dynamic and responsive approach to care would ensure that individuals have support that is appropriate to them at a particular time.

In the UK, clinical guidelines advise that those with diagnosed or suspected dementia should be referred to audiology services (National Institute for Health and Care Excellence, 2018), however the SCPs in this study reported that this was seldom the case in their experience. Other primary care services such as opticians, dentists and GPs seem to be embedded within care homes, in a way that audiology services are not. This results in barriers for people living with dementia accessing key aspects of hearing care that have the potential to improve management of their hearing loss and subsequently other health outcomes (Wells et al., 2020).

A lack of evaluation, and intervention, may exacerbate issues of misdiagnosis, as symptoms of hearing loss can be mistaken for dementia, and therefore go undetected. SCPs already have difficulty discriminating between the symptoms of the two conditions, leading to breakdowns in communication (Slaughter et al., 2014). Delaying people living with dementia access to appropriate support and care, for example by failing to address hearing loss could accelerate cognitive decline (Kricos, 2009; NHS England, 2017). It was the opinion of both SCPs and informal carers alike that regular assessments should be conducted within care settings to be able to implement appropriate management as soon as possible.

### Strengths and Limitations

This study reports an established approach to qualitative analysis, reported using an explicit and comprehensive method (O’Brien et al., 2014), ensuring rigor, transparency and trustworthiness. In qualitative research, data saturation may occur at an early stage of the process (Saunders et al., 2018); thus, sampling continues until there is sufficient data collected to provide a thorough explanation of the phenomenon in question. Despite a small sample size, data collection ceased when no additional themes were identified (Cleary et al., 2014; Saunders et al., 2018). By including key stakeholders, we explored an underdeveloped research area and advanced understanding of how hearing loss is managed in people with dementia in social care. By reporting findings from multiple perspectives, we were able to explore similarities and differences in perceptions of SCPs and informal carers (Kendall et al., 2009), for example with regard to hearing aid use. Moreover, this study provides new insights into the care provided regarding the management of hearing loss in people living with dementia in a particular context, that of social care.

An important limitation to acknowledge is that only one person with dementia and hearing loss took part in the online focus group; this approach to data collection may have precluded some people with dementia and hearing loss taking part. We recognise the importance of prioritising the meaningful inclusion of people with dementia in research. Future research in this area should endeavour to capture and include more views of people with dementia and hearing loss living in care settings, this may require a more inclusive approach than online focus groups. These may include adaptions and modifications such as using visual aids or prompts or the ability to participate in an interview rather than a focus group (Conway et al., 2023). Additional studies, using more inclusive methodologies, would be needed to determine the generalizability of our results to the larger population of people with dementia and hearing loss living in social care settings. In addition, the participants were mostly White British (n = 12), demonstrating limited ethnic diversity which limits the generalisability of the findings. We acknowledge that there may be distinct differences in the needs and experiences of individuals living with dementia, those with hearing loss, and their informal carers. Furthermore, a broader range of stakeholders for example audiologists and General Practitioners could be consulted in future research to ensure that other important issues relating to clinical care are not overlooked.

## Conclusions

The results of this research support the idea that formal, evidence-based training is required to support SCPs to optimise hearing care for people living with dementia. Our findings highlight that training should not only address technical aspects of hearing aid use and maintenance but also reflect the dynamic and progressive nature of both dementia and hearing loss. This would help ensure that SCPs are equipped to tailor hearing care to the ensuring that hearing support remains appropriate to evolving needs. In addition to training, the findings draw attention to systemic gaps in access to audiology services within social care settings. Future research should examine how audiology support can be more effectively integrated and embedded into social care pathways to support intervention and ultimately enhance quality of life for people living with dementia and hearing loss.

## Supplemental Material

Supplemental Material - Management of Co-existing Dementia and Hearing Loss in Social Care Settings: A Focus Group Study Supplemental Material for Management of Co-existing Dementia and Hearing Loss in Social Care Settings: A Focus Group Study by Emma Broome and Alice Green in Dementia

Supplemental Material - Management of Co-existing Dementia and Hearing Loss in Social Care Settings: A Focus Group Study Supplemental Material for Management of Co-existing Dementia and Hearing Loss in Social Care Settings: A Focus Group Study by Emma Broome and Alice Green in Dementia

## Data Availability

The data that supports the findings of this study are available from the corresponding author upon reasonable request.*
