# Supplementary material for: Management of Co-existing Dementia and Hearing Loss in Social Care Settings: A Focus Group Study
Source: Dementia (London). 2025 Jun 28;25(2):332–49. doi: 10.1177/14713012251356010 (PMC12816397; doi:10.1177/14713012251356010)
Supplement: Supplemental Material - Management of Co-existing Dementia and Hearing Loss in Social Care Settings: A Focus Group Study [file sj-pdf-1-dem-10.1177_14713012251356010.pdf]

Supplementary Material

Focus Group topic guide

Purpose:

We are interested in finding out more about how we can help support people living with dementia and hearing loss who access social care, including care homes, home care and day centres.

This research is funded by the National Institute of Health Research School for Social Care Research. And will provide the foundation for a larger body of work developing a tool to support people living with both dementia and hearing loss in social care.

We are interested in hearing from people with lived experience of dementia, and hearing loss, or both, and their carers. We are running a focus groups with social care staff, people living with dementia and/or hearing loss and their informal carers to gather their experience and perspectives as well.

Ethics

With your permission we are going to record the interview so that we can focus on what you are saying.

The focus group will last about an hour. If you need to take a break, please feel free to leave and come back or switch your camera off and come back.

We ask that you take it in turns to speak. So please try not to speak over each other.

If you have something you want to add, please use the raise hand function and we will make sure that you have an opportunity to speak.

We will encourage everyone to have an equal say. If some people haven't had a chance to speak, we may ask them a direct question.

The things you say in the interviews may be used in written reports, published articles and presentations including online but we will never use your name or any other information that may give away who you are.

Everyone's views are valid, please treat everyone with respect.

There are no right or wrong answers, people may well have differing points of view. We are interested to hear opposing views, please share your point of view even if it differs from what others have said.

Now I will turn on the recorder.

Focus Group Questions

Experience

Explore the experience relating to the care of people living with dementia and hearing conditions in social care settings.

Prompts for social care professionals:

- Please could you tell us about your background, where you work and the people you care for?
    - Information about setting e.g number of residents living with dementia, number living with hearing loss
- Could you describe any training you have had about dementia and/or hearing loss?
    - Did this include communication strategies?
    - What was most useful?
    - What was missing?
- Could you describe any specific strategies you have for providing care for people living with dementia and hearing loss?
    - How does this differ from routine care you provide?
    - What works well? What doesn't work as well?

Prompts for informal carers/people living with dementia and hearing loss:

- Please could you tell us about your experience as a person living with dementia and hearing loss/caring for someone living with dementia and hearing loss in social care
    - What kind of support do you/the person you care for receive to manage their hearing loss?
    - If applicable, how are you/the person you care supported to maintain hearing aids in social care settings?
    - How do you feel your/the person you care for, hearing-relating needs are monitored?
- What do you feel are the challenges in supporting people living with dementia and hearing loss in social care settings?
    - Relating to the care relationship
    - Relating to the social care environment (e.g. managing noise)

Ending

That is the end of the conversation.

Is there anything else you would like to tell us about that we have not already discussed?

Thank you very much for joining the focus group and for your valuable contributions.
