# Supplementary material for: Management of Co-existing Dementia and Hearing Loss in Social Care Settings: A Focus Group Study
Source: Dementia (London). 2025 Jun 28;25(2):332–49. doi: 10.1177/14713012251356010 (PMC12816397; doi:10.1177/14713012251356010)
Supplement: Supplemental Material - Management of Co-existing Dementia and Hearing Loss in Social Care Settings: A Focus Group Study [file sj-pdf-2-dem-10.1177_14713012251356010.pdf]

Manuscript: Management of co-existing dementia and hearing loss in social care settings: a focus group study

Standards for Reporting Qualitative Research Checklist (21-item checklist)

Developed from:
O'Brien BC, Harris IB, Beckman TJ, Reed DA, Cook DA. Standards for reporting qualitative research: a synthesis of recommendations. Acad Med. 2014;89:1245–51.

Index	Topic	Section (s)
**Title and Abstract**		
1	Title	Title Page
2	Abstract	Abstract
**Introduction**		
3	Problem formulation	Introduction
4	Purpose or research question	Introduction
**Methods**		
5	Qualitative approach and research paradigm	Introduction
6	Researcher characteristics and reflexivity	Procedure
7	Context	Procedure
8	Sampling Strategy	Participants and recruitment
9	Ethical issues pertaining to human subjects	Participants and recruitment
10	Data collection methods	Procedure
11	Data collection instruments and technologies	Procedure, Supplementary materials
12	Units of study	Participants and recruitment, procedure, Results
13	Data processing	Procedure, Data analysis
14	Data analysis	Data analysis
15	Techniques to enhance trustworthiness	Design, Participants, Data Analysis
**Results**		
16	Synthesis and interpretation	Results, Qualitative findings
17	Links to empirical data	Results, Qualitative findings
**Discussion**		
18	Integration with prior work, transferability, and contribution(s) to the field	Discussion
19	Limitations	Discussion
**Other**		
20	Conflicts of interest	Title Page
21	Funding	Title Page
